# Mechanically activated ion channel Piezo1 contributes to melanoma malignant progression through AKT/mTOR signaling

**DOI:** 10.1080/15384047.2022.2060015

**Published:** 2022-04-20

**Authors:** Simei Zhang, Shuang Cao, Mengyuan Gong, Wunai Zhang, Weifan Zhang, Zeen Zhu, Shuai Wu, Yangyang Yue, Weikun Qian, Qingyong Ma, Shengpeng Wang, Zheng Wang

**Affiliations:** a Department of Hepatobiliary Surgery, The First Affiliated Hospital of Xi’an Jiaotong University, Xi’an, Shaanxi, China; b Department of Nephrology and Medical Intensive Care, Charité – Universitätsmedizin, Molecular and Translational Kidney Research, Max Delbrück Center for Molecular Medicine in the Helmholtz Association, Berlin, Germany; c Cardiovascular Research Center, School of Basic Medical Sciences, Xi’an Jiaotong University Health Science Center, Xi’an, Shaanxi, China

**Keywords:** Piezo1, proliferation, metastasis, melanoma, AKT/mTOR signaling

## Abstract

Melanoma is a highly aggressive cancer that can metastasize at early stage. The aim of this study is to clarify the role of Piezo1 and its potential mechanism in regulating the malignant phenotypes of melanoma. In the present study, we first showed that Piezo1 was abnormally expressed in melanoma, which accelerated the malignant progression by activating AKT/mTOR signaling. Firstly, we found that Piezo1 was upregulated in melanoma and associated with poor survival. Additionally, Piezo1 knockdown significantly weakened intracellular calcium signal and viability of melanoma cells. Furthermore, Piezo1 knockdown inhibited the transendothelial migration and invasion in vitro, as well as metastasis in vivo. Mechanistically, we found that Piezo1 activated AKT/mTOR signaling to maintain malignant phenotypes of melanoma. Therefore, Piezo1 acts as an oncogene in melanoma cells and provides a novel candidate for melanoma diagnosis and treatment.

## Introduction

Malignant melanoma is one of the most aggressive cancers worldwide, with a plateaued incidence. In 2019, an estimated 96,480 new melanoma cases and 7 230 melanoma deaths were occurred in the United States.^1^ Melanoma is a highly aggressive tumor and exhibit early metastasis,^2^ which is one of the reasons for poor clinical outcomes.^3,4^ Therefore, there is an urgent need to unravel the molecular mechanisms of the malignant progression of melanoma, and provide a prospective therapeutic strategy.

Piezo1, is a trimeric, three-bladed propeller mechanosensitive channel protein consisting of three peripheral blade-like and a central cap-like structure.^5,6^ The Piezo1 ion channel protein is capable of sensing mechanical stimuli such as pressure, tension and fluid shear. In the activated state, it promotes calcium influx into cells.^7^ As a second messenger, intracellular Ca^2+^ can regulate cell migration, invasion, proliferation and gene transcription. In addition, Piezo1 plays a crucial role in embryonic development.^8^ The role of Piezo1 as an ion channel protein capable of sensing mechanical stimuli has been gradually investigated in tumors.^9,10^ However, it is unknown how Piezo1 could influence the malignant behavior of melanoma.

In the present study, we showed that Piezo1 was abnormally expressed in melanoma, which accelerated the malignant progression. We further found that Piezo1 regulated invasion, metastasis and cell cycle-related genes and activated AKT phosphorylation to control the viability, metastasis, invasion, and transendothelial migration *in vitro* and *in vivo*. Therefore, our study clarified the novel function of Piezo1 in melanoma cells and provided a novel candidate for melanoma diagnosis and treatment.

## Results

### Piezo1 is aberrant expressed in melanoma and increased intracellular Ca^2+^ concentration

**Figure 1.**
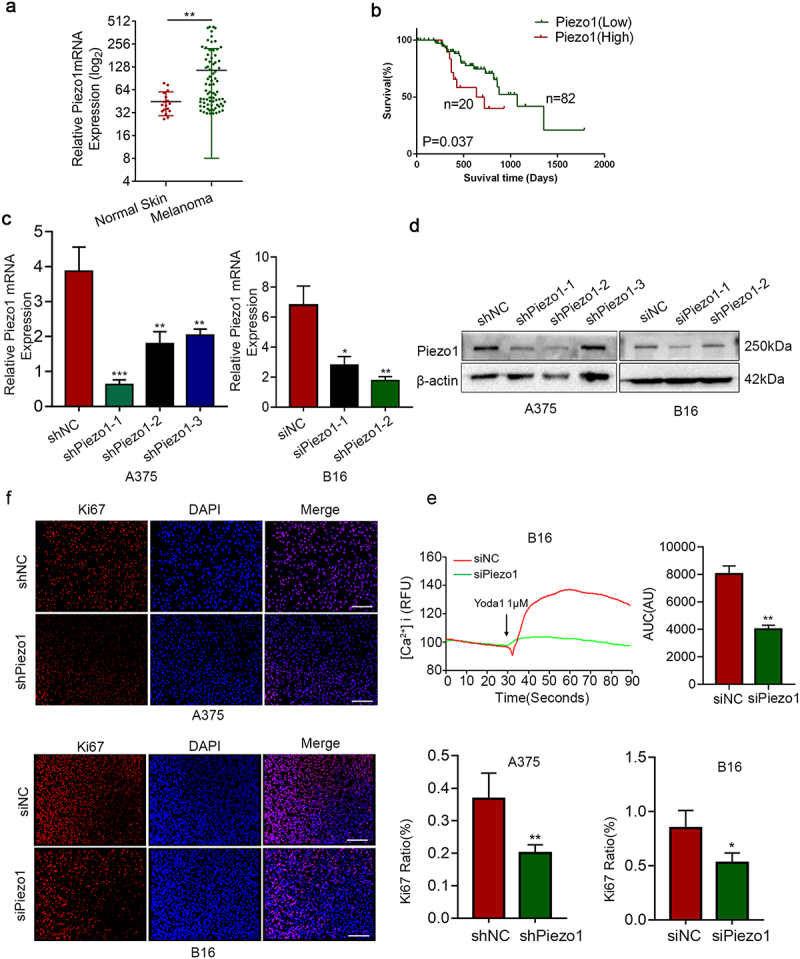
Piezo1 is aberrant expressed in melanom and increased intracellular Ca^2+^ concentration. **a** Piezo1 mRNA expression in normal skin (N = 17), melanoma (N = 73) tissues from GEO database (GSE46517). **b** Association between overall survival of melanoma patients and Piezo1 mRNA expression (best FPKM expression cut off value 12.5) from the TCGA database. **c**, **d** Quantitative real-time PCR and western blotting of Piezo1 mRNA and protein levels in A375, and B16 cells transfected with Piezo1 shRNA/siRNA (shPiezo1/siPiezo1) or negative control (NC). 18S and β-actin were used as loading controls. **e** Fluo-4-AM B16 cells (shNC, n = 30; shPiezo1, n = 36) were exposed to 10 μM Yoda1 and [Ca^2+^]_i_ was determined as fluorescence intensity (RFU, relative fluorescence units); Bar diagrams show the area under the curve (AUC) of the Ca^2+^-transient. **f** An Ki67 incorporation assay for staining proliferating cells (red indicates Ki67-incorporated cells, blue indicates nuclear staining with DAPI) was conducted in A375 and B16 cells; Bar diagrams showing the average of Ki67 to DAPI ratio in Piezo1 knockdown group cells and control group cells presented as the mean ± SEM (N = 7). Scale bars, 100 μm. Shown are mean values ± SEM.; *p < .05; **p < .01; ***p < .001.

According to the biology of melanoma, we first analyzed the expression level of Piezo1 in melanoma. Piezo1 mRNA expression level was upregulated in melanoma compared with normal skin tissues in TCGA database (Figure 1(a)). Additionally, human Protein Atlas website (https://www.proteinatlas.org) datasets showed that the upregulation of Piezo1 (best FPKM expression cut off value: 12.5) was correlated with the shorter overall survival of melanoma patients (Figure 1(b)). It seemed that the aberrant expression of Piezo1 could influence the malignant behavior of melanoma.

To confirm the role of Piezo1 in melanoma cells, we constructed relevant cells for the following experiments. The stable A375 cell line was established successfully with Piezo1 shRNA. Piezo1 knockdown of B16 was performed with (mouse)-Piezo1 siRNA, and the efficiency of knockdown was determined by qRT-PCR and western blot analysis (Figure 1(c,d)). Through intracellular calcium signal experiment, it showed that when control cells were stimulated with Yoda1 (an agonist of Piezo1, 10 μM), intracellular [Ca^2+^]_i_ recordings showed a transient increase in the free cytosolic Ca^2+^ concentration. Moreover, in the Piezo1 knockdown group, the intracellular calcium signal weakened significantly, and the enhancement of the signal was partly blocked (Figure 1(e)). The results suggested that the Piezo1 ion channel protein was functional in melanoma cells and modulated intracellular calcium concentration.

### Piezo1 promotes the malignant behavior of melanoma cells *in* *vitro*

**Figure 2.**
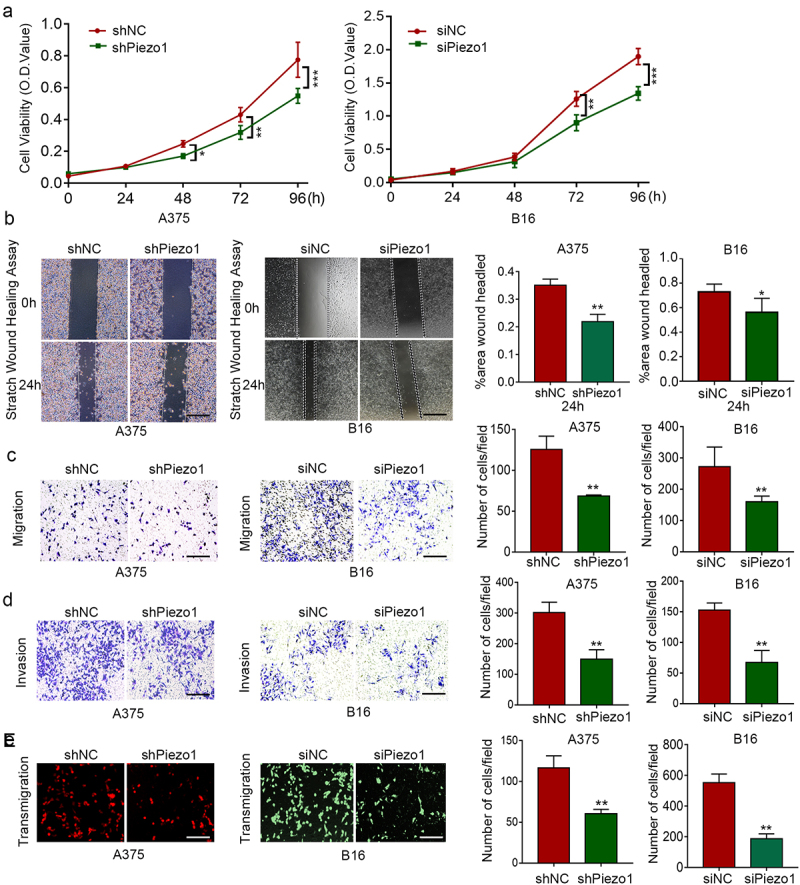
Piezo1 was oncogene in melanoma and increased the malignant behavior of melanoma cells. **a** Cell viability was detected by CCK8 assay with treatment as indicated. **b,c** Migration capacity was assessed by the wound healing assay and transwell assay in A375 and B16 cells. Quantification data showed on the right. Scale bars, 100 μm. **d** Invasion capacity was assessed by the transwell assay with Matrigel in A375 and B16 cells. Quantitative data showed on the right. Scale bars, 100 μm. **e** Transendothelial migration ability was assessed by the transwell assay in A375and B16 cells. Quantitative data showed on the right. Scale bars, 100 μm. Shown are mean values ± SEM.; *p < .05; **p < .01; ***p < .001.

The modulation of intracellular calcium signal could influence the cell biological behavior such as proliferation, invasion and metastasis, etc. To determine whether Piezo1 affected melanoma cell proliferation, a CCK-8 assay was applied to continuously monitor changes in cell viability at 0, 24, 48, 72, and 96 h. As shown in Figure 2(a）, knockdown of Piezo1 significantly suppressed the viability of melanoma cells from 48 h to 96 h (P < .05). Additionally, to observe the Ki67 staining change, there was lower Ki67 staining after Piezo1 knockdown (Figure 1(f)). Since DNA replication activity is usually considered to represent proliferation capacity and determined by cell staining with Ki67 antibody, these results indicated that Piezo1 inhibition could suppress melanoma cell viability.

Previous studies had clarified that intracellular calcium signaling was also associated with reorganization of the cytoskeleton such as F-actin, stress fiber and the formation of lamellipodia, which could play a vital role in regulating tumor invasion and metastasis.^3,11^ Our result confirmed that activation of the mechanically sensitive Piezo1 ion channel promoted inward calcium flow. Therefore, the potential role of Piezo1 in the migration and invasion capacity of melanoma cells was explored. The wound healing assay suggested that efficient knockdown of Piezo1 significantly impaired the wound healing rate compared with control group (Figure 2(b)). Additionally, transwell assay also proved that Piezo1 inhibition could influence cell migration ability (Figure 2(c)). Moreover, For the invasion capacity assay, it was shown that the number of invasive cells in shPiezo1 group were significant decreased than that in control group (Figure 2(d)).

To further confirm the influence of Piezo1 on the tumor cell metastasis ability, we applied the transendothelial migration assay *in vitro*. HUVECs suspended in Matrigel were seeded in the top chamber until they reached 100% confluence, and A375 and B16 suspended in FBS-free DMEM were seeded in the top chamber. After 12 hours, the upper layer of HUVECs and the tumor cells that had not crossed the endothelium were removed using a cotton swab. The results showed that the migration ability of Piezo1 knockdown cells crossing the HUVACs was significantly reduced (Figure 2(e)).

### Piezo1 exhibits oncogenic properties through modulation of AKT/mTOR

**Figure 3.**
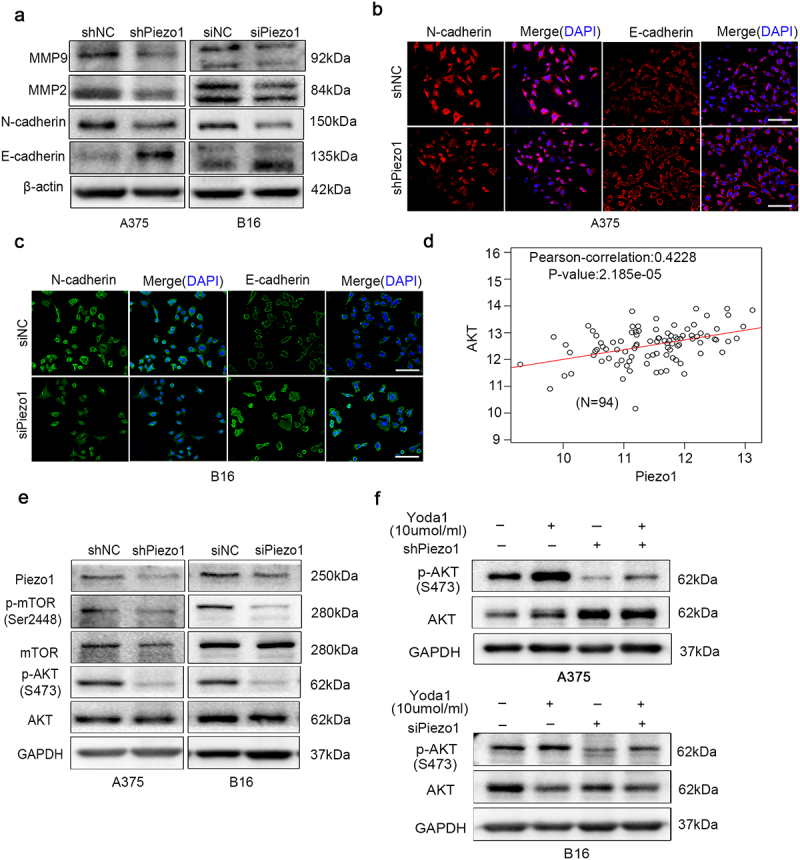
Piezo1 inhibition blocked malignant related genes in vitro through modulation of AKT phosphorylation. a The gene expression of E-cadherin, N-cadherin, MMP2 and MMP9 were detected by a western blot assay in A375 and B16 cells after Piezo1 knockdown. β-actin served as an internal control. **b,c** The results of immunofluorescence assay for N-cadherin, and E-cadherin expression were also determined in A375 and B16 cells transfected with Piezo1 knockdown, Scale bars, 100 μm. **d** The GEPIA online bioinformatics websites was used to analyze the relationship between the level of Piezo1 target genes and AKT pathway molecules. **e** Western blotting analysis of total and phosphorylated AKT, and mTOR in A375 and B16 cells after Piezo1 knockdown. GAPDH served as loading control. **f,g** Western blotting analysis of total and phosphorylated AKT, mTOR in A375 and B16 cells transfected with sh/siPiezo1 or sh/siNC after treatment with Yoda1 (A Piezo1 activator) for 24 h. GAPDH was used as a loading control.

As shown in Figure 3, there are a malignant behavior-related genes alteration after the knockdown of Piezo1. As to the cell cycle relation genes, the expression of CDK2 and cyclinD1 genes were significantly decreased after Piezo1 knockdown. However, P21 and PTEN, classical tumor suppressors were increased (Figure. （S1）). Numerous studies have suggested that epithelial-to-mesenchymal transition (EMT) contributes to early-stage dissemination of cancer cells and is pivotal for invasion and metastasis of melanoma.^12,13^ Matrix metalloproteinases (MMP2, MMP9) play vital roles in tissue remolding and cancer metastasis.^14^ As shown in Figure 3(a), mesenchymal-related gene (N-cadherin and E-cadherin), invasion-and metastasis-related gene (MMP2 and MMP9) expression in melanoma cells were significantly decreased after Piezo1 inhibition compared with control group. Similar results were also observed in the immunofluorescence assay, in which N-cadherin was downregulated and E-cadherin was upregulated in A375 and B16 cells after Piezo1 suppression (Figure 3(b,c)). Taken together, our observations indicated that Piezo1 was critical in malignant tumors events, including invasion and metastasis.

To further investigate the possible mechanism of Piezo1 on malignant behavior of melanoma, we analyzed several possible candidates that respond to mechanical biosignalling pathways by the online bioinformatics GEPIA websites and found that Piezo1 was associated with AKT (Figure 3(d)). Numerous evidences indicates that AKT/mTOR signaling is frequently activated in most malignant cancers, and regulates tumor cell proliferation, adhesion, survival, migration and invasion.^2,15–17^ In addition, AKT phosphorylation induces tumor cell migration and invasion through degradation of the MMPs-mediated matrix.^18^ Therefore, we treated melanoma cells with Yoda1 (a Piezo1 activator) at 10 μM as the most effective concentration. The results indicated that phosphorylation of AKT and mTOR were decreased in melanoma cells after knockdown of Piezo1 (Figure 3(e)). Furthermore, we treated melanoma cells with Yoda1 to investigate which pathways were associated with the activation of Piezo1. AKT phosphorylation was increased after the administration of Yoda1 which showed that AKT pathway was activated (Figure 3(f,g)). The above results indicated that the phosphorylation of AKT required the activation of Piezo1. Taken together, these data revealed that the level of activation of the mechanical ion channel Piezo1 increased the activation of the AKT/mTOR pathway.

### Piezo1 promotes the melanoma malignant behavior through the AKT/mTOR signaling pathway directly

**Figure 4.**
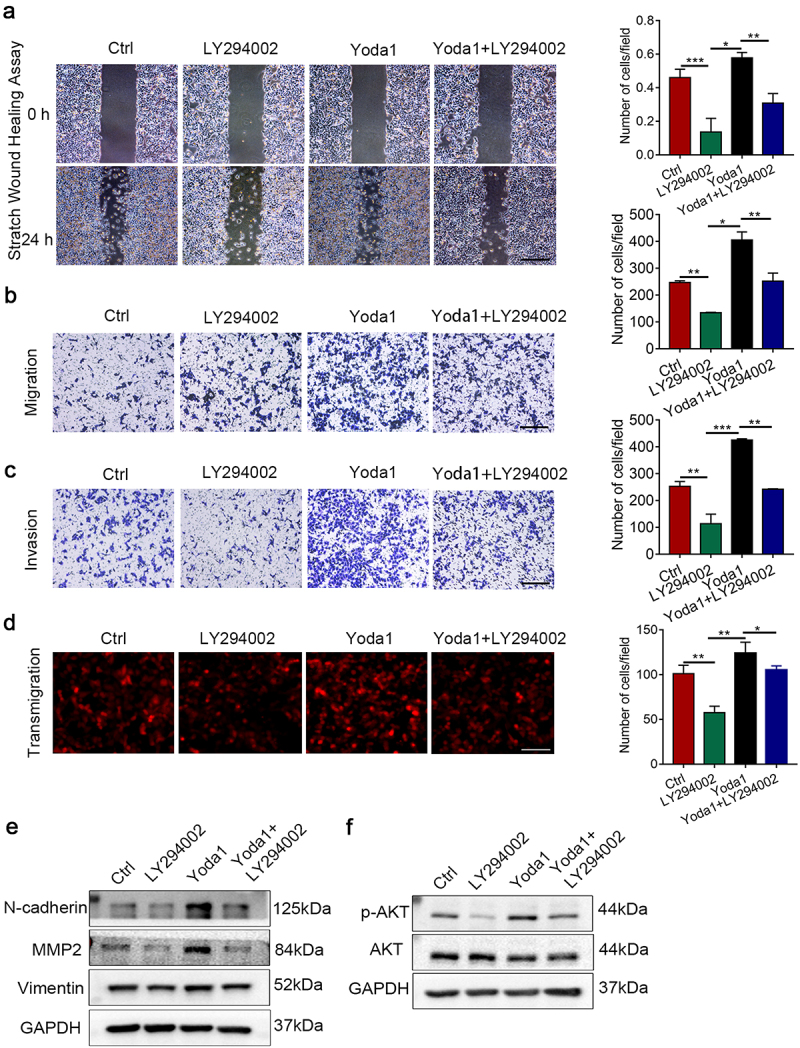
p

To further clarify whether the function of Piezo1 in regulating the malignant progression of melanoma was directly dependent on the PI3K/AKT/mTOR pathway, we treated wild-type A375 cells with the PI3K inhibitor LY294002, the Piezo1 activator Yoda1 and both. The blockage of PI3K/AKT/mTOR could significantly weaken the migration, invasion and transendothelial migration induced by Piezo1 activation with Yoda1 (Figure 4(a-d)). Moreover, as shown in Figure 4(e,f), p-AKT, MMP2, and N-cadherin protein levels were increased by the Piezo1 activator Yoda1, while LY294002 treatment reversed the expression of p-AKT, MMP2, and N-cadherin. These results collectively indicated that the oncogenic property of Piezo1 was mediated by activation of the PI3K/AKT/mTOR signaling pathway.

### Knockdown of Piezo1 attenuates melanoma metastasis and transendothelial migration *in* *vivo*

**Figure 5.**
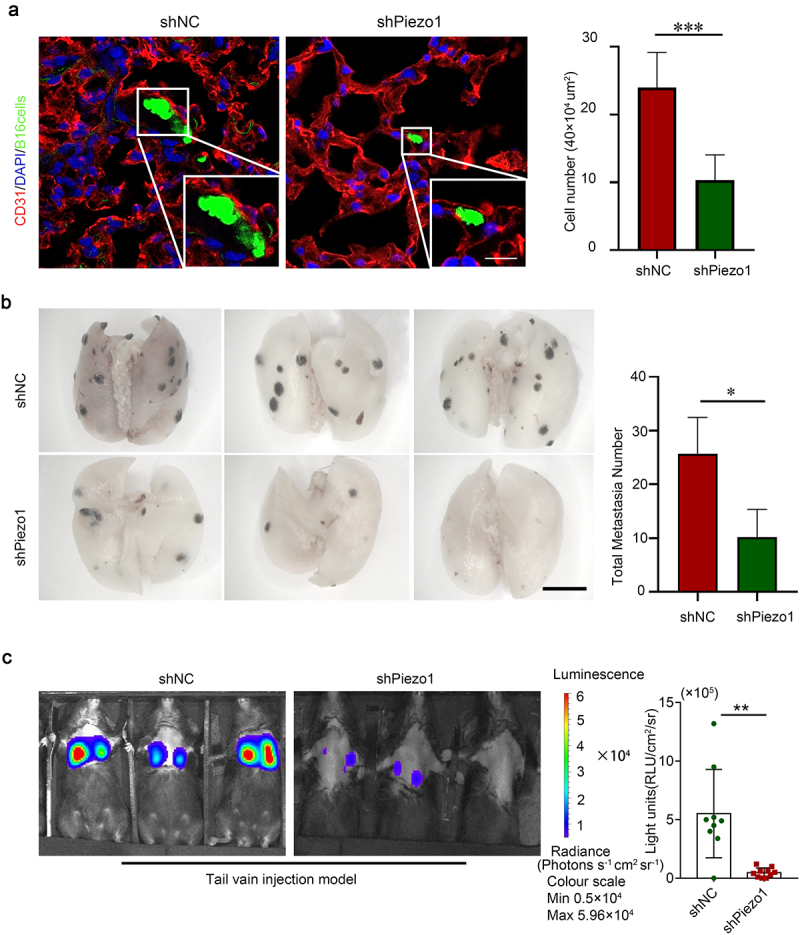
Knockdown of Piezo1 attenuated melanoma metastasis and transendothelial migration in vivo. **a** Immunofluorescence respectively after injecting B16 cells (shPiezo1and shNC fluorescently labeled) into mice for 6 hours (red indicated the blood vessel labeled with CD31, green indicated the cell with GFP, and blue indicated nucleus). Bar graph showed the number of cells extravasated into the vasculature. The scale bars, 20 μm. **b** The number of lung surface metastatic lesions from the shPiezo1 and shNC groups was calculated. Bar graph showing the average of the number of lung surface metastatic lesions. The scale bars, 10 mm. **c** Representative bioluminescence images from the shNC (N = 9) and shPiezo1 (N = 10) groups wereshown in the left panel. The scale showed the level of signal strength. Shown are mean values ± SEM.; *p < .05; **p < .01; ***p < .001.

Based on the results obtained from in vitro experiments, we verified the role of Piezo1 in vascular extravasation of tumor cells in vivo using the lung metastasis mouse model. By injecting B16 cells (shPiezo1and shNC fluorescently labeled) into mice and collecting lung tissue for frozen sectioning after 6 hours, we definitely found that the number of tumor cells which penetrated the blood vessels was 50% lower in the Piezo1-silenced group than that in the control group. (Figure 5(a)).

Moreover, the role of Piezo1 in melanoma was verified in vivo. We established a lung metastasis mouse model using the tail-vein injection model of B16 cell-lines. Two weeks later, the fluorescence signals of tumor cells in mice were tracked using a bioluminescence imaging system, and the results showed that there were less tumor signals in Piezo1-silenced tumor cells. Since melanoma appeared as black spots on lung tissue, the mice were sacrificed and lung tissue was collected. The results showed that the lungs of the shPiezo1 mice had only a few scattered black spots, while the control mice had evenly spaced spots (Figure 5(b,c)), p<.05). These data suggested that the Piezo1 channel could promote the metastasis of melanoma in vivo. Therefore, Piezo1 plays an important role in the transvascular migration of tumor cells and provides a basis for distant metastasis of the tumor.

## Discussion

Malignant melanoma is highly invasive and difficult to treat because of rapid malignant progression at an early stage. Therefore, the poor prognosis of melanoma has not improved.^19,20^ Hence, it is important to investigate the mechanism which cause the early malignant progression of melanoma. Here, we first demonstrated Piezo1 mechanosensitive ion channel in promoting tumor aggression in melanoma.

Alteration of mechanical properties is a physical hallmark of solid tumors, including melanoma. Increased tissue stiffness actively promotes malignant progression by affecting proliferation, migration, invasion, drug resistance, and growth-promoting signaling.^21,22^ Studies show that Piezo1 mediates breast cancer cell migration by regulating cell stiffness, contractility, and adhesiveness.^10,23^ In addition, Piezo1 has oncogenic effects in gastric cancer cells, including effects on cell migration, invasion and proliferation.^24^ In glioma, Piezo1 channels interact with integrin-FAK signaling and further promote tumor cell proliferation and tissue stiffness. However, it remains unclear how Piezo1 regulates malignant progression in melanoma.

In our study, we first identified the role of Piezo1 in melanoma in vitro and in vivo and clarified its underlying mechanisms. Piezo1 was widely overexpressed in melanoma cells, and it could promoted proliferation, invasion, metastasis, transendothelial migration and distant metastasis in vitro and in vivo. Our results showed that knockdown of Piezo1 suppressed the viability of melanoma cells and decreased the expression of Cyclin D1 and CDK2. Cyclin D1 and CDK2 are the key effectors in regulating cell cycle transitions. This result indicated that inhibition of Piezo1 expression caused cell cycle arrest, which was consistent with a previous study.^25^ Evidence suggests that fluid shear stress targets tumor bone morphogenetic and integrin proteins, which accelerates tumor cell cycle arrest.^26^ However, the relationship between fluid shear stress and Piezo1 has not been elucidated thus far.

Intracellular Ca^2+^, as a second messenger, mediates various cellular processes, including gene transcription, cell cycle, migration and invasion.^27^ Moreover, intracellular Ca^2+^ regulates the G1 to G2 transition, especially the G1 transition.^28^ Our results indicated that activation of Piezo1 increased in the free cytosolic Ca^2+^ concentration. In melanoma, intracellular Ca^2+^ release mediates tumor progression.^29^ Additionally, cell density increase when tissue stretching results in a sparse cell distribution, which activates Piezo1 and stimulates cell proliferation.^30^ These findings indicated that intracellular Ca^2+^ was associated with the melanoma malignancy. Although there is no direct evidence that intracellular Ca2^+^ regulates the AKT signaling pathway via Piezo1, study was proved that shear-induced calcium influx causes akt activation in endothelial cells.^31,32^ While the Piezo1 is capable of sensing mechanical stimuli such as pressure, tension and fluid shear. In the activated state, it promotes calcium influx into cells.^7^ Moreover, mitochondrial stress adaptation-increased intracellular levels of reactive oxygen species (ROS) and intracellular calcium are shown to induce alpha-amphiregulin (AREG) expression and secretion, while the up-regulation of AREG activates the PI3K/ Akt/mTOR pathway.^33^ Additionally, membrane stretch-mediated Ca2+ influx through Piezo1 is an important trigger for phosphorylation of AKT mediated upregulation of pulmonary arterial endothelial cells.^34^ Thus, in contrast to our results, intracellular Ca2+ may be regulate the AKT pathway in part via Piezo1 in melanoma cells.

We have focused on the interaction between the tumor mechanical microenvironment and biochemical signaling pathways. Our present study showed that Piezo1 knockdown inhibited invasion, metastasis, transendothelial migration, metastasis-related genes, including matrix metalloproteinases (MMP2, MMP9), and EMT (N-cadherin and E-cadherin). Next, we treated the cells with either LY294002 (PI3K inhibitor), or Yoda1 (Piezo1 activator) alone or in combination, and found that invasion, metastasis and transendothelial migration were activated and inhibited or partially activated, respectively. Previous studies reported that tumor tissue stiffness provides a mechanical microenvironment to activate Piezo1. Moreover, the expression of genes including Piezo1 involved in ECM remodeling can modulate tissue stiffness.^9,35^ Numerous studies have suggested that EMT could contribute to invasion and metastasis in melanoma.^36–38^ Moreover, tumor progression is dependent on extracellular matrix remodeling, fibroblast and macrophage activation and EMT.^39^ Since EMT is associated with the epigenetic signature of ECM remolding genes,^40^ we hypothesized that the metastatic process in melanoma cells is associated with Piezo1 regulation of ECM remodeling. A previous study showed that Piezo1 regulated the assembly of focal adhesion, activation of integrin signaling and expression of a number of genes involved in ECM remodeling, further increased tissue stiffness, which in turn activated Piezo1 and elevated the mechanosensory capacity of tumor cells.^9^

After discovering that Piezo1 is an oncogene that affects malignant progression, we further aimed to investigate the possible mechanism. Tumor cells respond to mechanical biosignalling pathways, including AKT/YAP-TAZ/β-catenin.^41^ Here, we found no difference in several candidates except the AKT pathway. Additionally, either a PI3K/AKT signaling pathway inhibitor or the Yoda1 (Piezo1 activator) or the combination of both reversed these phenomena. From this research, we demonstrated the knockdown of Piezo1 inhibited PI3K/AKT/mTOR pathway and that Yoda1-induced activation of Piezo1 increased PI3K/AKT/mTOR levels. Previous studies have revealed that the activation of AKT/mTOR has a crucial effect on the progression of various cancers.^16,18,25,42^ At present, some anticancer drugs targeting (PI3K/AKT/mTOR) signaling pathways have been developed and progressed to various stages of clinical trials, and some have proven to be effective.^43–45^ Our findings provided a possible therapeutic target for the malignant progression of melanoma.

In summary, we investigated the oncogenic role of Piezo1 in melanoma and demonstrated the mechanism which Piezo1 regulated malignant progression of melanoma through the PI3K/AKT/mTOR signaling pathway. According to the function the Piezo1 mechanosensitive ion channel in melanoma, it may be a potential target for drug designed against cancer progression.

## Methods

### Cells and reagents

The human A375 melanoma cell line, mouse B16 melanoma cells, and human umbilical vein vascular endothelial cell (HUVEC) were purchased from the American Type Culture Collection (ATCC, US). All cell lines were cultured in Dulbecco’s modified Eagle’s medium (DMEM; Gibco; Thermo Fisher Scientific, USA) with 10% fetal bovine serum at 37°C aired with 5%CO_2_. The following antibodies were used in this study at the indicated dilutions for western blot (WB) and immunofluorescence (IF): Piezo1 (Ca-15939-1-AP, 1:1000 for WB), MMP2(10373-2-AP,1:1000 for WB), and beta-actin (66009-1-1 g, 1:2000 for WB) were purchased from Proteintech. Ki-67(9129, 1:400 for IF), AKT (4691, 1:1000 for WB), p-AKT (Thr308, 13038, 1:1000 for WB), E-cadherin (3195, 1:1000 for WB), N-cadherin (13116, 1:1000 for WB), and vimentin (5741, 1:1000 for WB) were purchased from CST (Cell signaling Technology, Inc). MMP9 (ab38898, 1:1000 for WB) was purchased from Abcam.

### siRNA /shRNA and lentiviral transduction

siRNAs were purchased from Qiagen. At cell density of 1–2 × 10^4^/cm^2^, 50 pmol of siRNA was mixed gently with RNAiMAX in 100 μl of Opti-MEM, incubated for 30 min at room temperature and added to 1.5 ml of cell culture medium (DMEM). Cells were incubated with the complexes for 8–12 h at 37°C in a CO_2_ incubator and thereafter, the medium was replaced with complete medium (DMEM plus 10% FBS). For RNA and protein experiment, cells were harvested 24 h and 48 h posttransfection, respectively. The siRNA target sequences directed against RNAs encoding Piezo1 were 5’-CACCGGCATCTACGTCAAATA- 3’ and 5’-TCGGCGCTTGCTAGAACTTCA-3’. Piezo1 knockdown mouse melanoma cells were named siNC and siPiezo1.

Piezo1 knockdown lentivirus and scramble control were purchased from GeneCopoeia (Guangzhou, China). The target sequence for Piezo1 shRNA was GGTCTACAAGATTGTCTACAT, and the negative control sequence was GCTTCGCGCCGTAGTCTTA. Transfection was carried out using lentiviral particles and polybrene according to the manufacturer’s protocol. Cells were selected with puromycin (4 μg/ml) for 10 days. Stable Piezo1 knockdown human melanoma cells were named shNC and shPiezo1. The effect of gene silencing was analyzed by real-time PCR and western blot.

### Determination of intracellular [Ca^2+^]

To determine of the intracellular Ca^2+^ concentration, cells were placed in 96-well microplates for fluorescence-based assays (Invitrogen) and loaded with Fluo-4 AM (Molecular Probes, Life Technologies). Live-cell images were acquired with an Olympus IX81 microscope. Fluorescence intensity was measured with a FlexStation 3 (Molecular Devices).

### Immunofluoresence

Cells plated on coverslips were fixed with 4% paraformaldehyde for 30 min, permeabilized with 0.2% Triton X-100 for 10 min, and blocked with 5% bovine serum albumin (Sigma-Aldrich, Germany) for 1 hour. Then the cells were incubated overnight with a primary antibody of Ki67. Next, the samples were extensively washed with PBS buffer, incubated with fluorescein isothiocyanate-conjugated AffiniPure goat anti-rabbit IgG secondary antibody (dilution, 1:200; cat, EF00002, SPARK JADE) for 60 min and stained with DAPI (cat, D1306,1:5000, Invitrogen) for 5 min in the dark. Laser scanning confocal microscopy (Nikon A1R/A1) was used to observe the samples.

### Cell viability assay

A375 and B16 cells were seeded in 96-well plates at 1000 cells and 200 μl per well, respectively, and incubated overnight in 10% FBS medium. After 24 h, 48 h, 72 h, and 96 h, 20 μl of CCK-8 (Dojindo Molecular Technologies, Inc., Kumamoto, Japan) was added to 180 μl of completed medium per well, and the absorbance was determined at 490 nm. The data are the result of three independent experiments.

### Wound healing assay and transwell assay

The A375 and B16 melanoma cell lines were seeded on 6-well plates, and when the cells reached 100% confluence using, a 200 μl pipette tip was used to scratch the cells to create artificial wounds. Wound healing was observed by inverted microscopy after 24 h.

Transwell invasion assays were performed in 24-well plates. Transwell chambers (pore size, 8.0 μm; Millipore, Billerica, USA) were coated with Matrigel (BD Bioscience, Oxford, UK). A375 and B16 (300 μl, 5 × 10^4^ cells per well) suspended in DMEM containing free-FBS were seeded in the top chamber, and 600 μl medium containing 10% FBS was placed in the lower chamber. After 24 h, cells which penetrate to the lower surface of the chamber were fixed with paraformaldehyde for 20 min and then stained with 0.1% crystal violet for 10 min. Cell migration and invasion were determined by counting the stained cells under a light microscope in 5 randomly selected fields.

### Transendothelial migration assay

Tumor cell transendothelial migration assays were performed using transwell chambers that were coated with Matrigel 8 hours in advance. HUVECs were seeded in the top chamber until they reached 100% confluence, and A375 and B16 (300 μl, 6 × 10^4^cells per well) suspended in FBS-free DMEM were seeded in the top chamber, and 800 μl medium containing 10% FBS was placed in the lower chamber. After 12 hours, the upper layer of HUVECs and the tumor cells that had not crossed the endothelium were removed using a cotton swab. Then 50 μl and 250 μl of 1× PBS were added to the upper and lower layers, respectively, and the tumor cells were counted under fluorescence microscopy.

### Quantitative real-time PCR assay (qRT-PCR)

Total RNA was isolated with RNAfast 200 reagents (Fastagen Biotechnology,Shanghai, China). After quantitation by absorbance at 260 nm, 1000 ng of the total RNA was reverse transcribed by PrimeScript RT Master Mix (Takara Bio, Dalian, China). Quantitative PCR was carried out using SYBR-Green PCR Master Mix (Takara Bio, Dalian, China) with the following specific primers: Piezo1(human), F: cgtcttcgtggagcagatg, R: gcccttgacggtgcatac; Piezo1(mouse), F: ggaaaagagctccgacacac-3 R: ccaggacttccccacctatt; 18S, F: cagccacccgagattgagca, R: tagtagcgacgggcggtgtg.

### Western blot analysis

Cells were lysed in RIPA buffer (50 mM Tris, pH 8.0,150 mM NaCl, 0.1% SDS, 1% NP40 and 0.5% sodium deoxycholate) supplemented with protease inhibitor (1% inhibitors cocktail and 1 mM PMSF) (Roche Applied Science, Germany). Lysates were centrifuged at 12000 g for 15 min at 4°C, subjected to 8% SDS-PAGE and transferred to polyvinylidene fluoride (PVDF) membranes. Membranes were incubated with the indicated primary antibody at 4°C overnight and were developed using the ECL chemiluminescent detection system (BioRad, USA). The experiment was repeated three times.

### In vivo cell extravasation assay

Piezo1-silenced B16 melanoma cells were suspended in 1× PBS and stained with 1 μg/ml CFSE(Invitrogen, US) for 15 min at 37°C. Cells were then washed with PBS, suspended in complete medium and incubated for 30 min at 37°C. Then, cells (1.5 × 10^5^, 100 μl) were suspended in PBS and introduced into the mice by tail-vein injection. After 6 hours, mice were sacrificed and lung tissues were analyzed by immunofluorescence (IF)^46^to label the vascular endothelial cells of the lungs and observe the tumor cells inside and outside the blood vessels.

### In vivo metastasis tumor model

The tail-vein injection metastasis model was generated as described in previous studies. Wild-type (WT) (C57/BL6J) mice (8 weeks) were used according to protocols approved by the Ethics Committee of Xi’an Jiaotong University. Piezo1-silenced B16 melanoma cells with luciferase markers (1.5 × 10^5^ cells, 100 μl) were suspended in serum-PBS and introduced into the circulation of mice by tail-vein injection. After 6 weeks, 150 mg/kg D-luciferin substrate (Biosynth, Naperville, IL, USA) in PBS was injected into the abdominal cavity. Fifteen to 20 min later, bioluminescence imaging (BLI) was performed to detect distant metastases in the lung and other organs after the mice were anesthetized. The mice were then sacrificed, the lungs were lavaged with PBS to clear them, and the number of melanoma metastases was counted under a microscope (Invitrogen, USA).

### Bioinformatics and statistical analyses

The GSE46517 dataset was used to analyze the expression of Piezo1 in normal skin, primary melanoma and metastatic melanoma. The overall survival-related Piezo1 expression was analyzed from the Human Protein Atlas website (https://www.proteinatlas.org/). The Kaplan–Meier method was used to estimate the survival, and the log-rank test was used to assess the difference. The GEPIA online bioinformatics analysis websites (http://gepia.cancer-pku.cn/) was used to analyze the relationship between the levels of Piezo1 target genes and AKT pathway molecules. The data were analyzed and graphed using GraphPad Prism version 7.0 software (GraphPad Software, USA). Comparisons between multiple groups (≥ 3) were performed using one-way ANOVA. Other statistical analysis were performed using two-tailed Student’s t-tests. P values<.05 were considered statistically significant.

## Supplementary Material

Supplemental Material
