# Supplementary figures and images for: Mechanically activated ion channel Piezo1 contributes to melanoma malignant progression through AKT/mTOR signaling
Source: Cancer Biol Ther. 2022 Apr 20;23(1):336–47. doi: 10.1080/15384047.2022.2060015 (PMC9037449; doi:10.1080/15384047.2022.2060015)

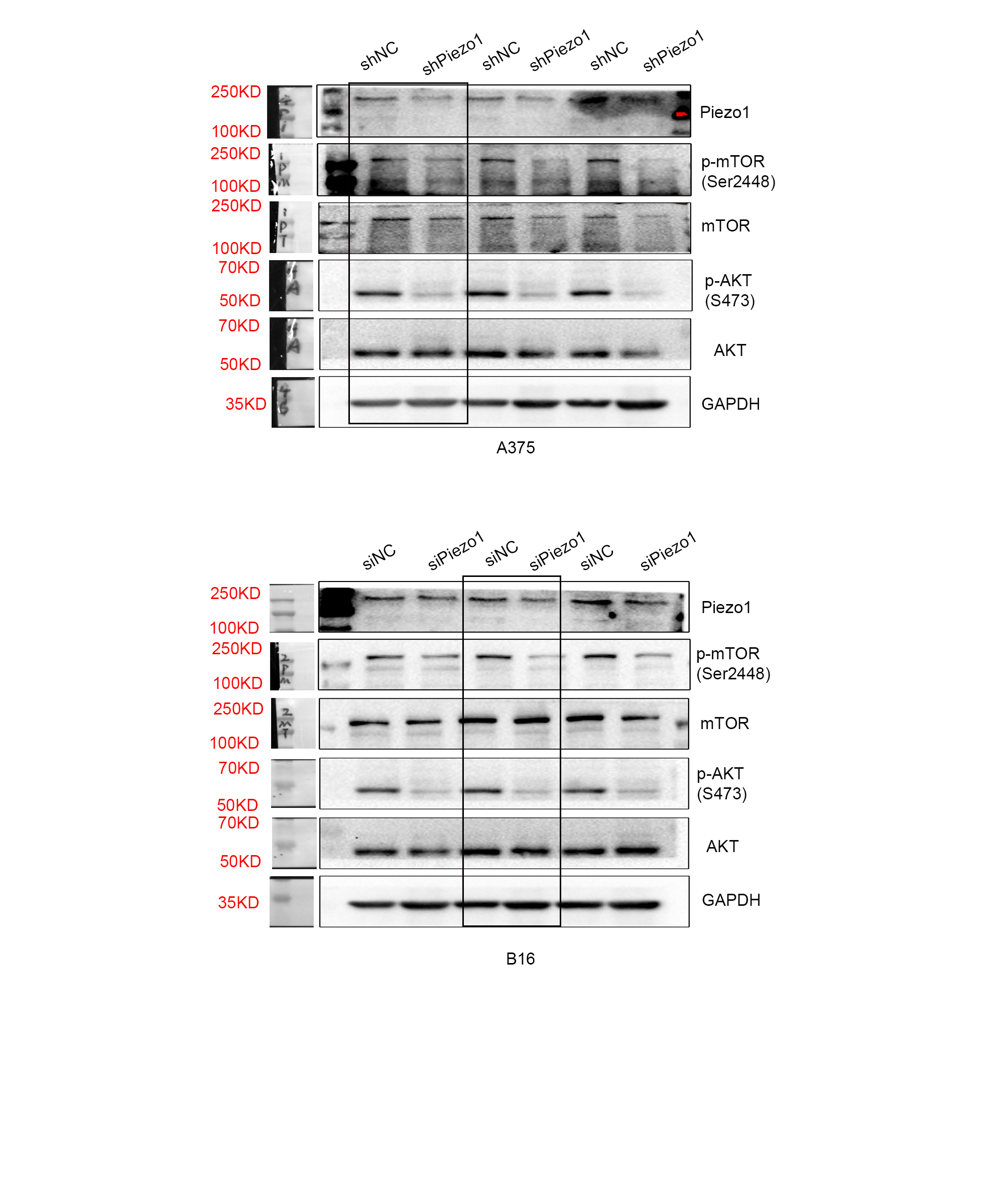

Supplement: Supplemental Material [file KCBT_A_2060015_SM4143.zip › Fig 3e.tif]

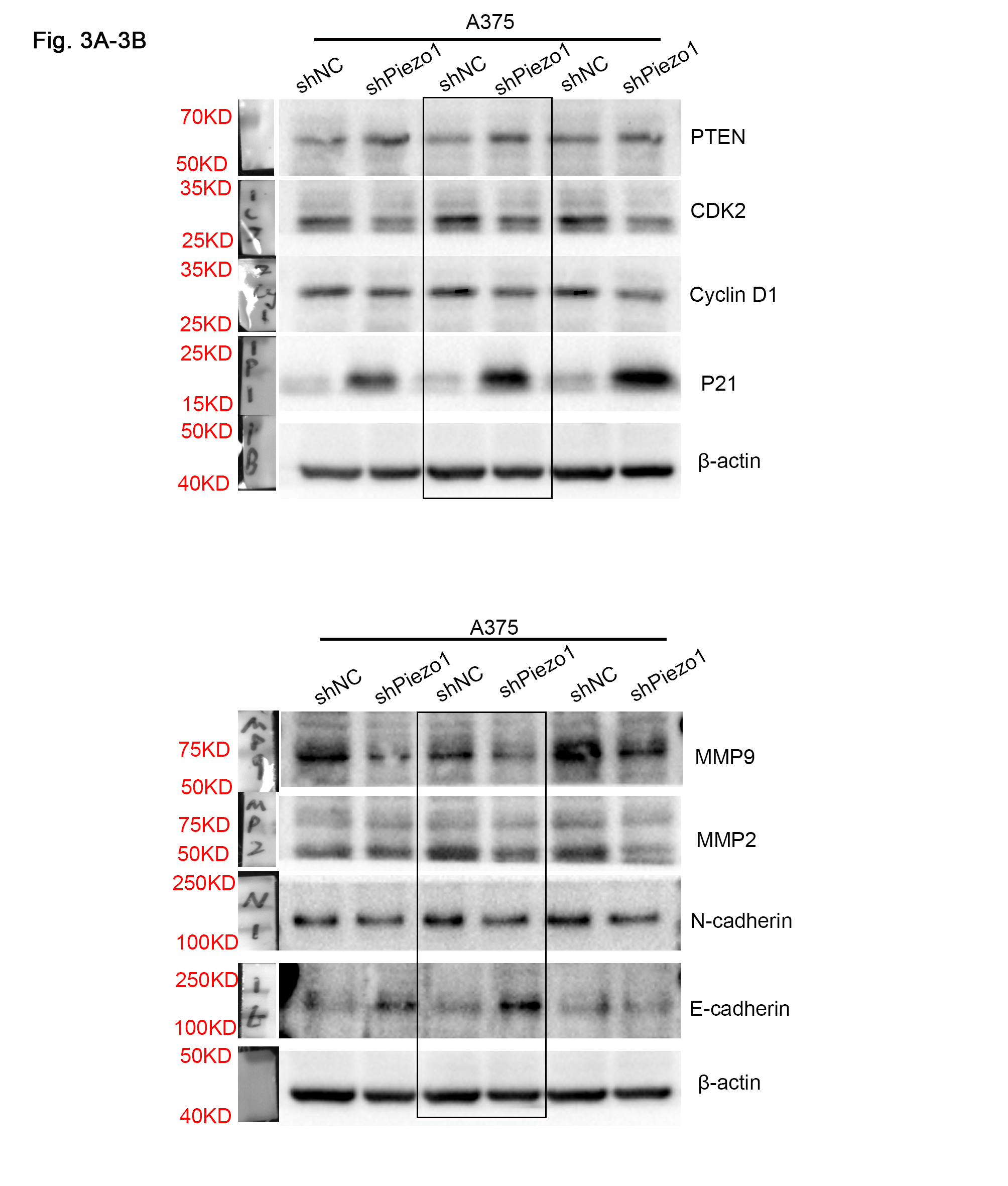

Supplement: Supplemental Material [file KCBT_A_2060015_SM4143.zip › FIG.3A(A375)-1.tif]

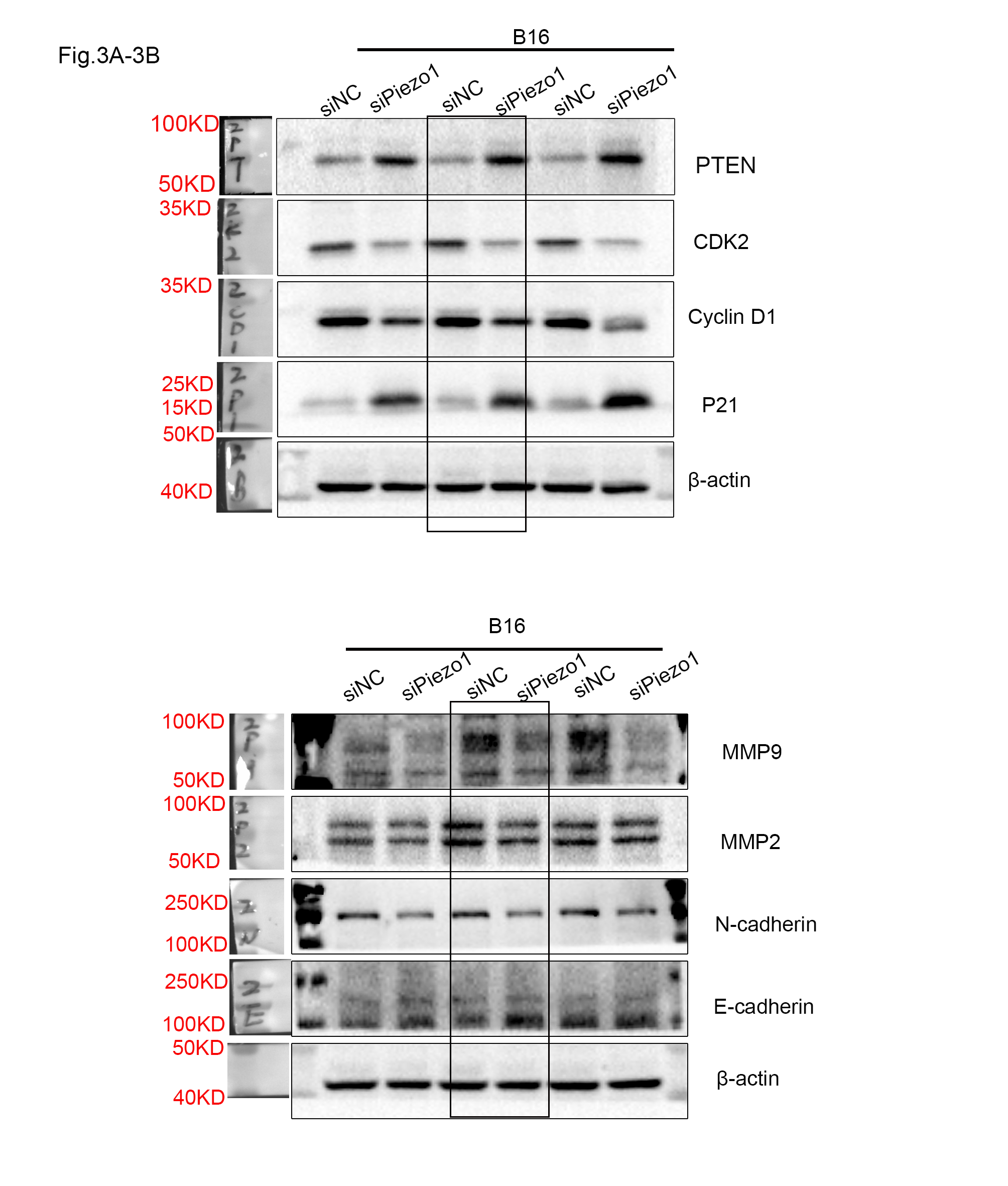

Supplement: Supplemental Material [file KCBT_A_2060015_SM4143.zip › FIG.3A(B16)-1.tif]

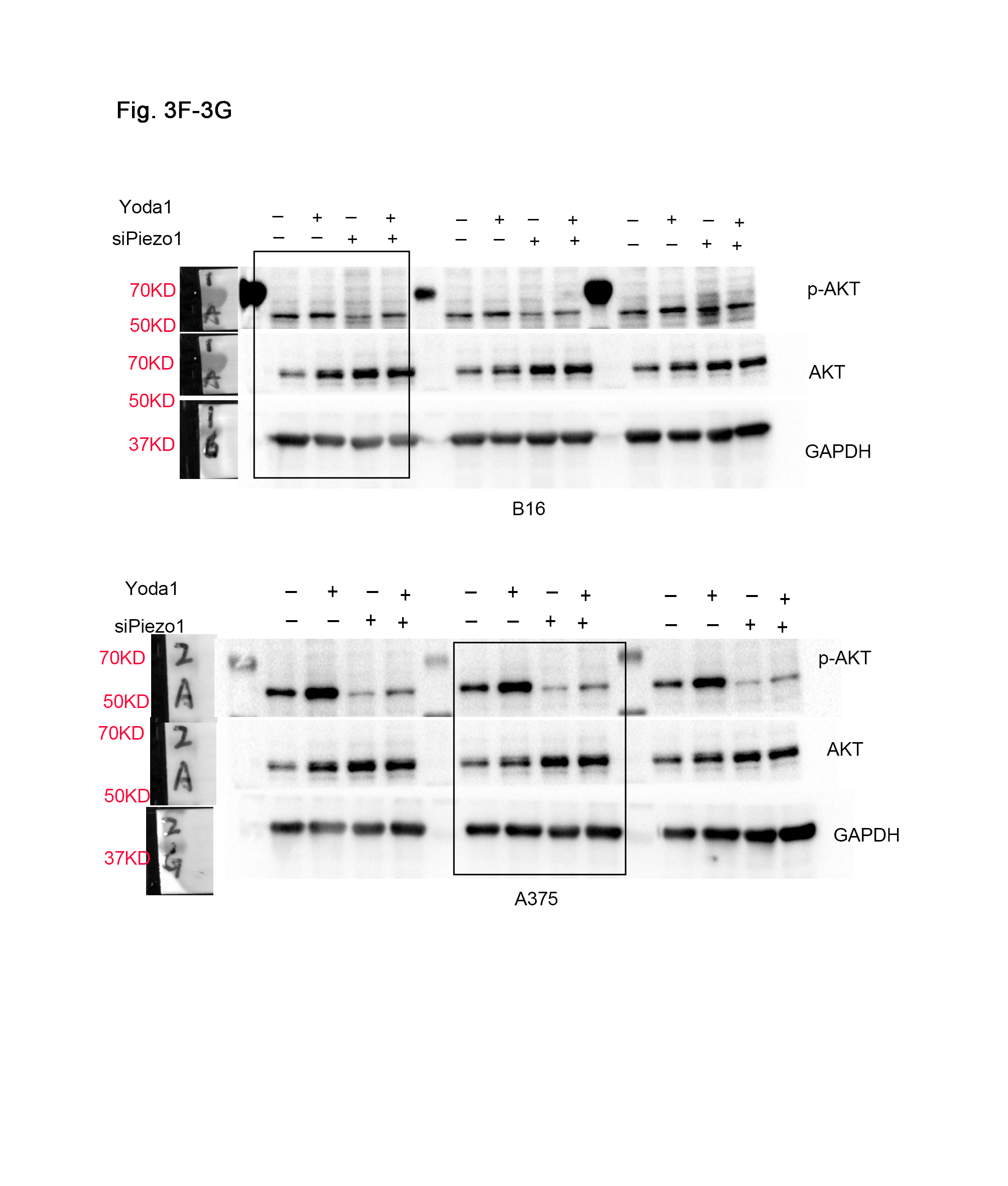

Supplement: Supplemental Material [file KCBT_A_2060015_SM4143.zip › Fig3f-g.tif]

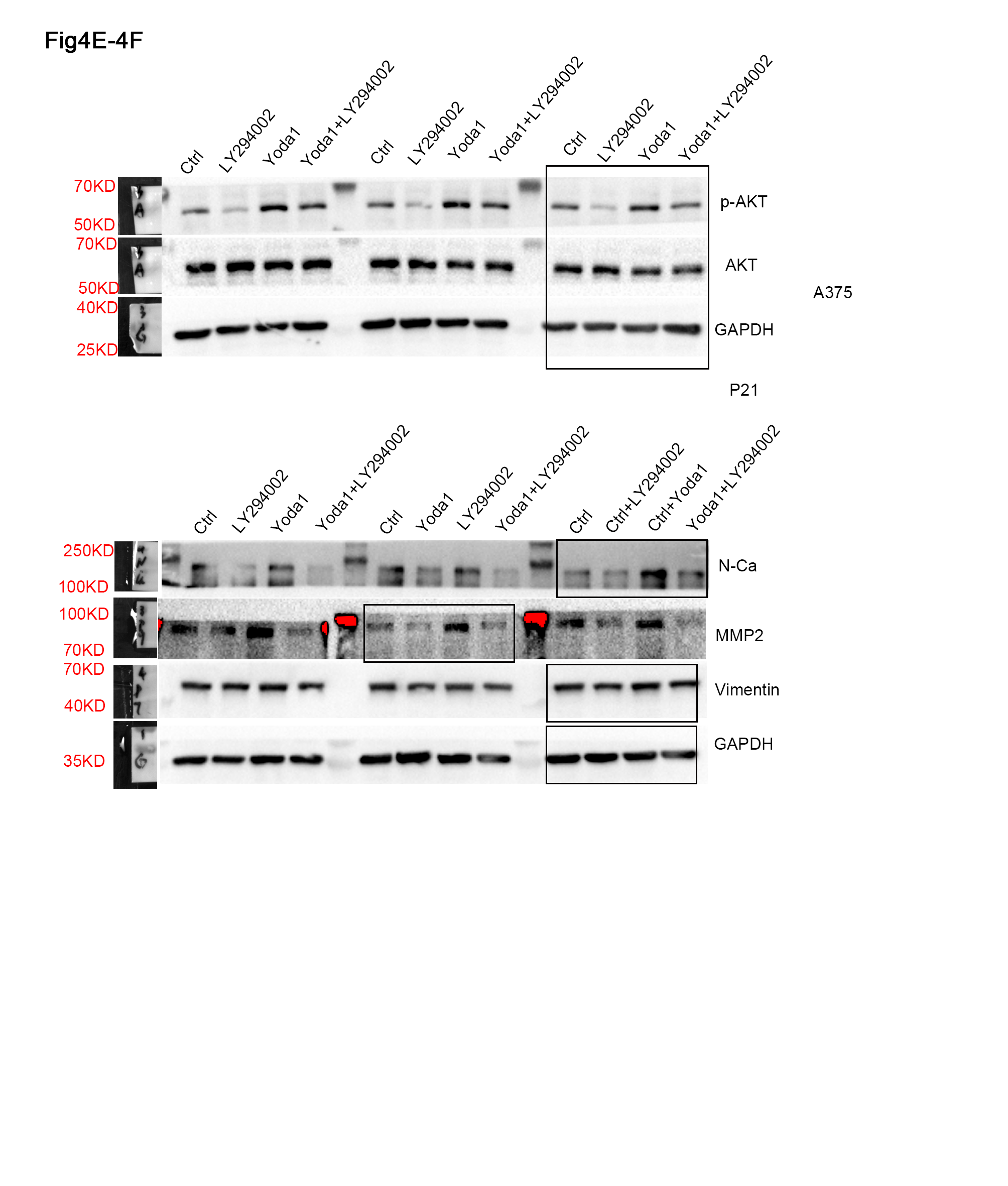

Supplement: Supplemental Material [file KCBT_A_2060015_SM4143.zip › Fig4e-g.tif]

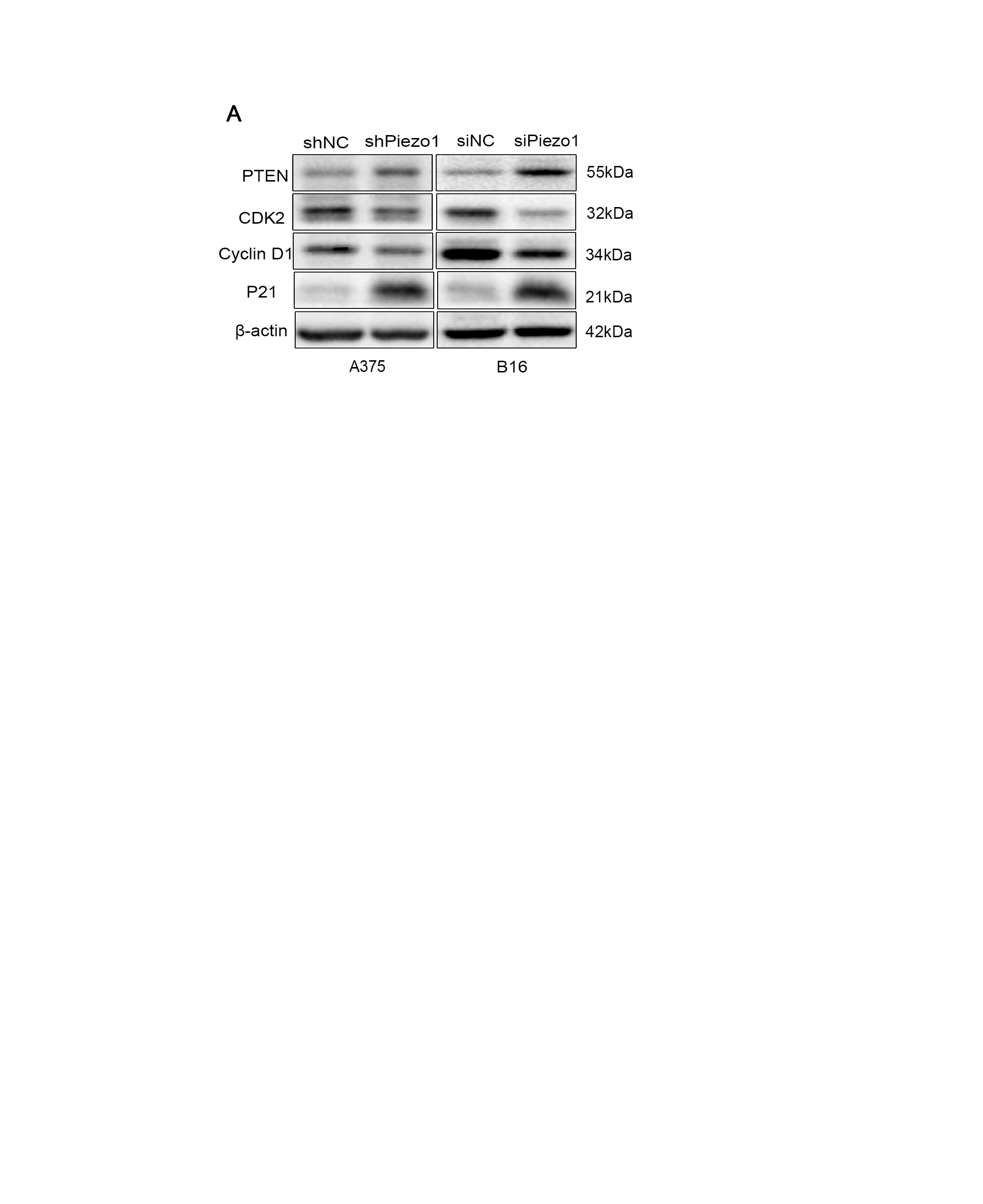

Supplement: Supplemental Material [file KCBT_A_2060015_SM4143.zip › supplementary 1tif.tif]

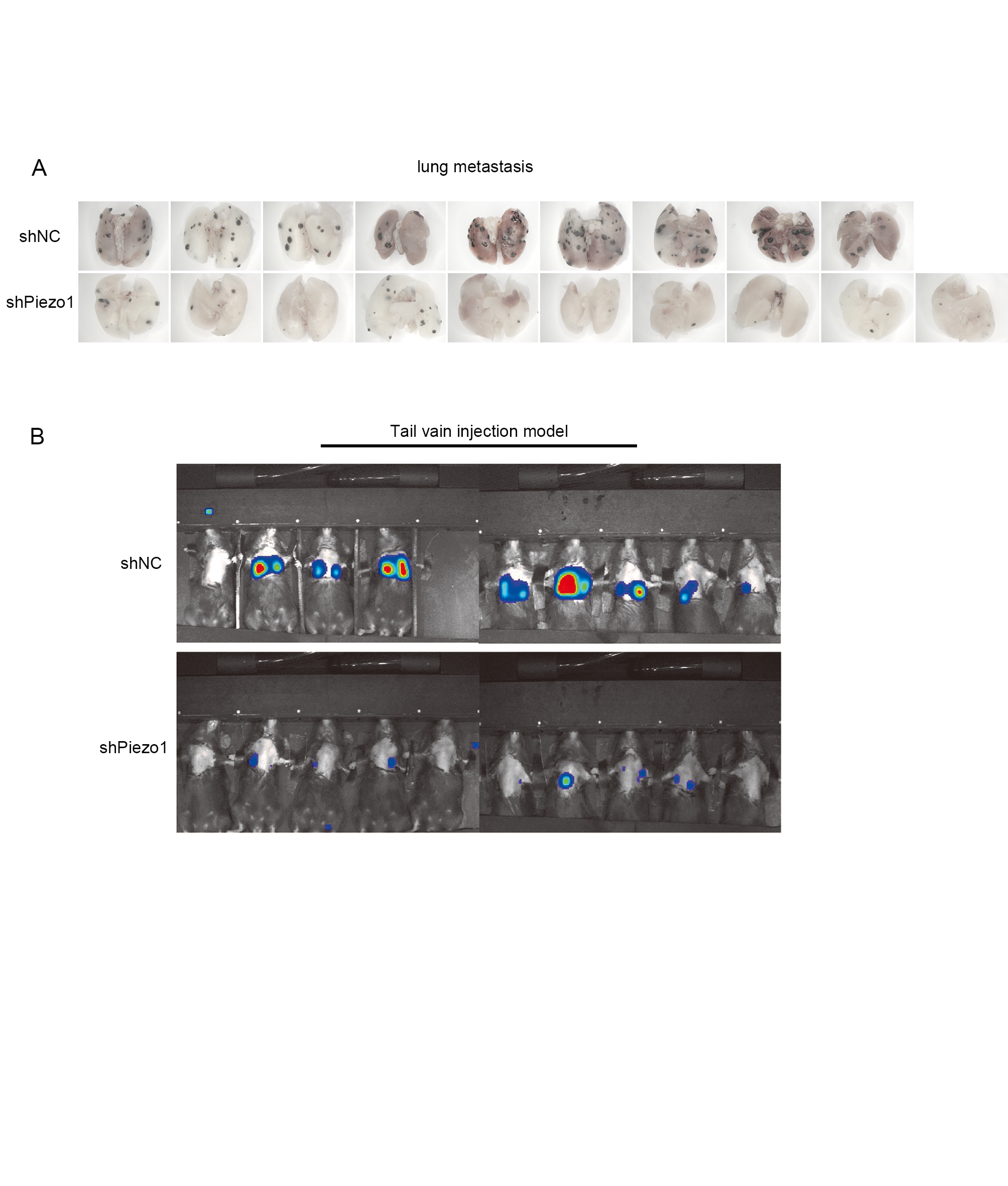

Supplement: Supplemental Material [file KCBT_A_2060015_SM4143.zip › supplementary 2 .tif]
